# Neighbourhood deprivation and adolescent self-esteem: Exploration of the ‘socio-economic equalisation in youth’ hypothesis in Britain and Canada

**DOI:** 10.1016/j.socscimed.2013.02.021

**Published:** 2013-08

**Authors:** James H. Fagg, Sarah E. Curtis, Steven Cummins, Stephen A. Stansfeld, Amélie Quesnel-Vallée

**Affiliations:** a MRC Centre of Epidemiology for Child Health, UCL Institute of Child Health, 30 Guilford Street, London WC1N 1EH, UK; b Institute of Hazard and Risk Research and Wolfson Research Institute, Dept. of Geography, Durham University, South Road, Durham DH1 3LE, UK; c Healthy Environments Research Programme, School of Geography, Queen Mary University of London, Mile End Road, London E1 4NS, UK; d Centre for Psychiatry, Old Anatomy Building, Barts and The London, Charterhouse Square, London EC1M 6BQ, UK; e McGill University, International Research Infrastructure on Social Inequalities in Health (IRIS), 1020 Pine Av. West, Montreal QC, Canada H3A1A2; f McGill University, Department of Sociology, 1020 Pine Av. West, Montreal QC, Canada H3A1A2; g McGill University, Department of Epidemiology, Biostatistics and Occupational Health, 1020 Pine Av. West, Montreal QC, Canada H3A1A

**Keywords:** Neighbourhood deprivation, Britain, Canada, United States, Health inequalities, Geographical inequalities, Self-esteem, Adolescent, Equalisation, Psychological health

## Abstract

Material deprivation is an important determinant of health inequalities in adults but there remains debate about the extent of its importance for adolescent wellbeing. Research has found limited evidence for an association between adolescent health and socio-economic status, leading authors to suggest that there is an ‘equalisation’ of health across socio-economic groups during the adolescent stage of the life-course. This paper explores this ‘equalisation’ hypothesis for adolescent psychological wellbeing from a geographical perspective by investigating associations between neighbourhood deprivation and self-esteem in Britain and Canada. Data from the British Youth Panel (BYP) and the National Longitudinal Survey of Children and Youth (NLSCY) on adolescents aged 11–15 for the time period 1994–2004 were used to estimate variations in low self-esteem between neighbourhoods using multilevel logistic regression. Models were extended to estimate associations between self-esteem and neighbourhood deprivation before and after adjustment for individual and family level covariates. Moderation by age, sex, urban/rural status, household income and family structure was investigated. There were no significant differences in self-esteem between the most deprived and most affluent neighbourhoods (Canada unadjusted OR = 1.00, 95% CI 0.76, 1.33; Britain unadjusted OR = 1.25, 95% CI 0.74, 2.13). The prevalence of low self-esteem was higher (in Canada) for boys in the *least deprived* neighbourhoods compared to other neighbourhoods. No other interactions were observed. The results presented here offer some (limited) support for the socio-economic equalisation in youth hypothesis from a geographical perspective: with specific reference to equalisation of the relationship between neighbourhood deprivation and self-esteem and psychological health in early adolescence. This contrasts with previous research in the United States but supports related work from Britain. The lack of interactions with key social and economic variables suggests that findings might apply across a range of family circumstances and different communities in Britain and Canada. Policy implications are discussed.

## Highlights

► The relationship between adolescent self-esteem and neighbourhood deprivation in Britain and Canada was studied. ► Self-esteem was not associated with neighbourhood deprivation in large, national representative British and Canadian cohorts. ► Findings contrast with previous United States studies examining adolescent self-esteem and neighbourhood deprivation.

## Introduction

Socio-economic inequalities in health are found almost ubiquitously across the world and across the life course (Marmot et al., 2010). These inequalities have been clearly demonstrated in adults and young children for groups distinguished by education and household income (Emerson, Graham, & Hatton, 2005) and area level deprivation (Riva, Gauvin, & Barnett, 2007). The area disparities are potentially important from a population health perspective because if health inequalities are associated with area level characteristics, intervention at the small area level might be an important means to ‘level up’ population health inequalities in a cost-effective way (Marmot et al., 2010). However, socio-economic inequalities in adolescent health are not so universally observed, with reviews of the empirical evidence for a range of health outcomes and markers of socio-economic status suggesting that socio-economic inequalities in childhood equalise in adolescence (West, 1997; West & Sweeting, 2004), only to reemerge in later life. This paper seeks to extend and develop the evidence base for this hypothesis to the family and area level using data from nationally representative surveys of adolescents in Britain and Canada.

## Background

Mental health and wellbeing among young people is an area of research that has been receiving increasing attention in recent years. Recent policy documents in Britain have placed greater emphasis on mental health, such as ‘No health without mental health’ (Department of Health, 2011). This strategy aims to take a life course perspective to protect and promote mental health and wellbeing and to reduce inequalities among adults, children and young people. Until recently, there has not been widespread recognition of broader mental health issues among adolescents and research has generally focussed on conduct disorders and disruptive behaviour. Relatively little research has investigated area level inequalities in adolescent mental health and wellbeing and even less research has examined inequalities in self-esteem. Global self-esteem is defined here as an evaluative attitude towards the self (Rosenberg, 1979) that is an integral component of health and wellbeing (Bradshaw & Keung, 2011) and closely related to other mental health outcomes such as depression (Harter, 1999).

Relationships between adolescent self-esteem or psychological health and neighbourhood deprivation might be expected for three reasons. Firstly, recent work has demonstrated that there is considerable instability in the self-esteem and psychological health of individual adolescents over time (Baldwin & Hoffmann, 2002; Greene & Way, 2005), suggesting that self-esteem might not be as immutable to environmental intervention as previously believed. Secondly, work which draws upon the classic sociological theory of *reflected appraisal* (Mead, 1934), suggests that individuals internalise the opinions of others in their own self-evaluations; and this has been used to hypothesise that neighbourhood reputation may explain associations between neighbourhood deprivation and self-esteem (Haney, 2007; Macintyre, Maciver, & Soomans, 1993). Finally, adolescents in more deprived neighbourhoods might perform less well on average on educational outcomes (Jencks & Mayer, 1990) and this might be attributable to a lack of resources and opportunities for adolescents at the neighbourhood level. However, an adolescent in a deprived neighbourhood might attribute their performance to individual competence rather than neighbourhood constraints, contributing to a lower sense of self-worth. This is summarised by Cutrona, Wallace, and Wesner (2006) in her discussion of the relationship between neighbourhood deprivation and psychological health, where she argues that people “often do not realise that they are affected by the context around them and thus mistakenly blame themselves” (p. 188).

Thus there are clear theoretical paths linking self-esteem and psychological health more generally with deprivation at the neighbourhood level. To our knowledge, two research studies investigate empirically the relationship between self-esteem (the outcome which we investigate here) and neighbourhood deprivation in adolescence (Drukker, Kaplan, Feron, & van Os, 2003; Turley, 2002). In both studies, self-esteem was found to be inversely associated with neighbourhood deprivation, independently of individual and family characteristics. Similar studies investigating associations with depressive symptoms supported these conclusions, finding positive associations between depressive symptoms and neighbourhood deprivation (Schneiders et al., 2003; Wickrama & Bryant, 2003). We note that both of the nationally representative studies (Turley, 2002; Wickrama & Bryant, 2003) which found relationships between self-esteem or internalising psychological outcomes and neighbourhood deprivation were based in the United States. Furthermore, several reviews have reported extensive evidence for relationships between neighbourhood poverty and other youth health outcomes (Jencks & Mayer, 1990; Leventhal & Brooks-Gunn, 2000; McBride Murry, Berkel, Gaylord-Harden, Copeland-Linder, & Nation, 2011).

The theoretical and empirical work reviewed above suggests that there should be strong reasons to expect inequalities at the area level in adolescent self-esteem and internalising mental health outcomes such as depression and anxiety. However, work from Britain has suggested that socio-economic inequalities which are observed throughout early to late childhood appear to ‘equalise’ or even reverse in gradient in early adolescence, before re-emerging in late adolescence and early adulthood (West, 1997; West & Sweeting, 2004). This ‘socio-economic equalisation’ hypothesis has been theorised to be a result of the influence of the increasing importance of social status and social relations with peers, relative to socio-economic status of families which drive child and adult inequalities (West, 1997).

The socio-economic equalisation in youth hypothesis was developed in Britain through a review of studies examining associations between parental social class citing, for example, the classic Isle of White (in Britain) studies (Rutter, Maughan, Mortimore, & Ouston, 1979) which showed no associations between ‘neurotic disorders’ and parental social class. Although evidence for the utility of parental social class measures in research on equalisation has been disputed (Judge & Benzeval, 1993), evidence for the hypothesis has also been demonstrated in relation to area deprivation. For example, West and Sweeting (2004) provide an analysis of adolescents in the West of Scotland Study supporting equalisation by area deprivation: they report reverse gradients such that ‘malaise’ symptoms increased with decreasing quintiles of area deprivation. More recent research in Britain supports their findings when investigating relationships between neighbourhood deprivation and emotional health (Collishaw, Goodman, Ford, Rabe-Hesketh, & Skrondal, 2009) and psychological distress (Fagg, Curtis, Stansfeld, & Congdon, 2006). These studies showed that there was no relationship between neighbourhood deprivation and the psychological health outcomes under study.

In broad terms, the United States, Canada and Britain are culturally and economically similar, sharing national strategic and economic interests as founder members of the Organisation for Economic Cooperation and Development (OECD, 2011). However, the United States is recognised to be distinct from Canada and Britain in terms of income inequality, (Mair, Diez-Roux, & Galea, 2008; Ross et al., 2000), social welfare and health care provision, and the high levels of absolute poverty and crime in its most deprived neighbourhoods (Marmot, 1998; Oreopoulos, 2008).

The current evidence base in adult depression (Mair et al., 2008), and from the review of primary studies above, suggests that neighbourhood deprivation may be strongly associated with low self-esteem in the United States, but less differentiated in relation to neighbourhood deprivation in Britain. As studies in the United States are nationally representative while British studies have been regional to date, we suggest that nationally representative British data are needed to strengthen the evidence base for this apparent difference between the two countries. We also note that no studies are available from Canada to extend the socio-economic equalisation theory at the neighbourhood level to that national context. We argue that Canada is more similar to Britain than to the United States in the factors which might strengthen inequalities between neighbourhoods (such as income inequality, absolute neighbourhood deprivation and social welfare provision). Therefore we theorise that low self-esteem in adolescence may also, as in Britain, equalised across levels of neighbourhood deprivation in Canada.

This paper reports an empirical analysis which extends the equalisation hypothesis in three ways. First, using self-esteem as an outcome; second, by considering processes operating at the small area level, and third by strengthening empirical work from the British national context and extending work to the Canadian context. Thus, the main aim of the study was to assess whether or not associations between individual and neighbourhood level socio-economic status supported the equalisation hypothesis – with a particular focus on neighbourhood deprivation. Secondary aims were to establish if associations were found consistently across social groups and wider geographical factors, and if patterns of association were, as theorised, similar in Britain and Canada.

## Data and methods

### Datasets and derivation of analysis samples

**Fig. 1:**
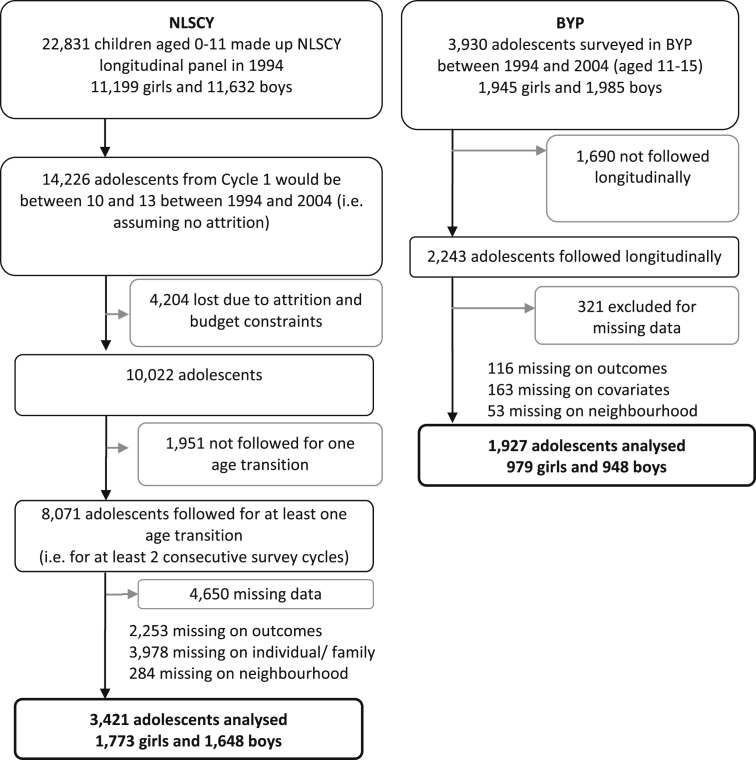
Sample derivation of NLSCY and BYP samples.

This study employs two large scale longitudinal surveys, from Britain and Canada. The British Youth Panel (BYP) consists of the adolescent children of members of the British Household Panel Survey (BHPS). The BHPS is an annual panel survey of a nationally representative sample of more than 5000 households in the United Kingdom (described in Taylor, 2009). The British Youth Panel (BYP) was added to the BHPS in wave 4 (1994) and includes approximately 700 household members aged 11–15 years at each wave. Each year, newly eligible 11 year-olds (those who turn 11 by December 1st) are included in the BYP, using a rotating panel design (Gayle, Lambert, & Murray, 2009), and followed annually until they turn 16. This paper examines information from children, their parents and other household members surveyed in waves 4 to 13 corresponding to the period 1994–2004 (see Fig. 1). Data from Northern Ireland were excluded in this analysis as information about neighbourhood characteristics could not be linked in to the individual dataset, (the nature of the funding used for BHPS data collection in Northern Ireland meant that different rules applied for the release of data) thus the analysis is representative of adolescents living in Britain only, not the UK.

The National Longitudinal Survey of Children and Youth (NLSCY) employs an accelerated cohort design with the overall aim of investigating the health and development of Canadian children from birth (0 years) to early adulthood (25 years). The survey follows children and adolescents aged 0–11 years in 1994 on a biennial basis (1994 *N* = 22,831). The sampling frame included all Canadian children in 1994 excluding those living in the Yukon or Northwest Territories, individuals living in institutions, and those living on Indian Reserves (StatCan, 2006). Data from adolescents, their parents and households are used from cycles 1 (1994) to 6 (2004), and cover the same period (1994–2004) as the BYP (see Fig. 1).

### Outcome: low self-esteem

Self-esteem is measured in the BYP using a 5-item scale. The items are similar in content and style to the Rosenberg (1979) self-esteem scale and aim to measure global self-esteem. Respondents are asked whether they agree strongly (4), agree (3), disagree (2) or disagree strongly (1) with statements like: ‘I feel I have a number of good qualities’ and ‘At times I feel I am no good at all’ (see Electronic Appendix 1 for further items). Three items had a negative valence and were recoded before all five were summed - the scale thus ranged from 5 to 20 (20 equates to high self-esteem) and was internally homogeneous with an alpha coefficient of 0.71.

The four item self-esteem scale used in the NLSCY was the General Self-Scale (Marsh & O'Neil, 1984). This scale was originally developed as part of a wider self-concept inventory but the other items were not measured in the NLSCY (apart from the peers subscale described below in the covariates section). Respondents were asked whether they felt that statements like “In general, I like the way I am” were “False” (0), “Mostly false” (1), “Sometimes false/sometimes true” (2), “Mostly true” (3), “True” (4). All the statements (see Electronic Appendix 1 for further items) had a positive valence and so were simply summed with no recoding. The scale ranges from 0 to 16 with an alpha coefficient of 0.80. The NLSCY scale was highly skewed and no transformation (including square root, log 10, natural log or inverse) resulted in an approximation of a normal distribution.

As the NLSCY scale was skewed, both the NLSCY and the BYP outcomes were dichotomised and the odds of reporting low self-esteem were modelled. In the absence of published validated cut-offs for low self-esteem on either scale, a statistical cut-off of one standard deviation below the median of each scale was used. This was chosen to allow qualitative comparison of models fitted on both surveys.

The items used in both scales and their relationship to the long form of the Rosenberg Self-Esteem questionnaire are described in more detail in Electronic Appendix 1.

### Neighbourhood deprivation

British neighbourhood deprivation is operationalised using the Townsend Index, devised by Townsend (1987) and commonly used to measure deprivation at the small area level. The score comprises four measures: unemployment as a percentage of those aged 16 years and over who are economically active; the percentage of all households who do not own a car; the percentage of all households who do not own their own home; and household overcrowding (where overcrowding is the 1991 definition of more than 1 person per room). Values for unemployment and overcrowding are transformed using the natural log to take into account skew in the statistical distribution of these variables. All four components were then standardised to the same scale using the *z*-score method before being summed into a composite index. Townsend deprivation scores were calculated for all wards (England and Wales) and postcode sectors (Scotland), comprising 10,506 overall, and ranging in population size from 54 to 31,609 (mean = 5222, median = 4307) across England, Wales and Scotland. Quintiles of neighbourhood deprivation were then created, defined relative to the deprivation ranking of all wards and postcode sectors in England, Scotland and Wales prior to being linked into the survey.

Neighbourhood deprivation is operationalised in the NLSCY using the material deprivation index proposed by Pampalon and colleagues (2000). The Pampalon Index comprises information on the population aged over 15 years: proportions without a high school certificate or diploma; proportions unemployed and average income. It is meant to reflect average levels of financial and economic poverty in the local population. Neighbourhood deprivation information was matched to individual level data in the NLSCY using specifically developed methods for this survey (Gonthier, 2009). The area data relate to Dissemination Areas (DAs), the smallest geographical areas for which Canadian census data are disseminated. There were 52,993 DAs in Canada nationally in the 2001 census, ranging in population size from 400 to 700 people.

Both deprivation indices were designed to operationalise the latent construct of neighbourhood deprivation as conceptualised by Townsend et al. (1987) in Britain and Canada. However, it is important to note that the scales are measured 10 years apart and at different geographical scales which may lead to differences in the relationships between deprivation and self-esteem in Britain and Canada. This limitation, and findings from the sensitivity analyses designed to address it, are outlined in the discussion section.

### Covariates

We measured demographic, social, socio-economic and geographic characteristics of individuals and neighbourhoods which were expected to covary with neighbourhood deprivation and low self-esteem. The variables used are described below. As relationships with families and friends were measured differently in both surveys, more comprehensive descriptions are also available in Electronic Appendix 2.

#### Sex, age and visible minority status

Sex and age were recorded in both surveys. In the BYP, children were measured every year at 11 through 15 years old; the NLCY measurement was biennial with children aged 10–11 in the first wave, 12–13 years in the second and 14–15 years in the third. Ethnic minorities are clearly a socially and culturally heterogeneous ‘group,’ however their visible minority status (distinguished by skin tone) could be considered a marker for racial discrimination which might have a bearing on self-esteem (McBride Murry et al., 2011). In the BYP, adolescent ethnicity was not measured directly, but adolescents were classed as ‘visible minorities’ where at least one of their parents self-identified as being from Black, mixed, or Asian heritage as opposed to those from White European heritage. A similar marker was used in the NLSCY. In the NLSCY, this variable was derived by asking adolescents, ‘To which cultural group did your ancestors belong’ and they self-identified as White European (Canadian, French, English, German, Scottish, Irish, Italian, Ukranian, Dutch, Jewish, Polish, Portuguese), Visible Minority (South Asian, Black, Chinese, Other) and First Nations (Metis, Inuit/Eskimo, North American Indian). The latter were distinguished as they have a distinct psychosocial wellbeing profile associated with environmental dispossession (Richmond & Ross, 2009). This is thought to have expression in the high prevalence of a wide range of social problems such as alcohol and substance abuse, teenage pregnancy and long-term, intergenerational unemployment in these groups in contemporary Canadian society (Richmond & Ross, 2009).

#### Relationships with parents

In the BYP, parent–child relationships were summarised by two, three-category variables relating to frequency of arguments and frequency of talking about close things. These were based on four items, where the first two related to the mother–child relationship; how often do you talk about close things with your mother and how frequently do you argue with your mother. The available responses were: 1 – hardly ever, 2 – less than once a week, 3 – more than once a week, 4 – most days, and 5 – no mother. These questions were repeated with respect to the adolescent's father. The responses to all four items were conflated to 1 (hardly ever), 2 (less than/more than once a week) and 3 (most days) to facilitate interpretation and 5 was considered missing. As single parent families generally had only one valid value, only one response could be used, so where responses were available for both parents, the most positive response was used. The NLSCY carried two scales relating to adolescents' perceptions of their relationships with their parents measuring parental nurturance and parental rejection (described fully in Schaefer, 1965).

#### Happiness with family and family functioning

Happiness with family was assessed in the BYP with reference to the request that adolescents “ tick the box that best describes how you feel about your family” with responses ranging from 1 (completely happy), 4 – don’t know, to 7 (completely unhappy). Categories 1–3 were conflated to “Happy with family” and categories 4–7 were collapsed to “Unhappy/don't know” owing to the small proportion who reported unhappiness with family (3%). This was the closest marker of family functioning that could be found which was available at all waves of the BYP. In the NLSCY, family functioning was measured using the 12-item general subscale of the McMaster Family Assessment Device (described fully in Miller, Epstein, Bishop, & Keitner, 1985).

#### Happiness with friends

Happiness with friends was assessed in the BYP in the same way as happiness with families (described above). Categories were collapsed to “Happy with friends” and “Unhappy/Don't Know” as with the family item. In the NLSCY, peer relationships are measured using the peer relations subscale (described in full by Marsh & O'Neil, 1984).

#### Maternal education

To represent maternal education in the BYP a four level variable was used, consisting of less than GCSE or equivalent, GCSE equivalent, A-level equivalent and Diploma/Degree level. In the NLSCY, a three level variable was created consisting of Less than High School, High School, and Diploma/Degree level.

#### Maternal depression

The 12 item General Health Questionnaire was used in the BYP to measure maternal distress/depression. Presence or absence of distress was defined as a binomial variable using the cut-off described by Goldberg and Williams (1988). In the NLSCY the short form of the Centre for Epidemiologic Studies Depression scale (Radloff, 1977) was used and a distressed state was defined using the cut-off described by Somers and Willms (2002).

#### Family structure

In both the NLSCY and the BYP, family structure was categorised as living with both biological parents, living with a single parent (biological or otherwise), or living in a reconstituted family (one biological parent, one step parent).

#### Urbanicity

Urbanicity was considered to be an important moderator of relationships between neighbourhood deprivation and mental health relationships following previous work (Riva, Curtis, Gauvin, & Fagg, 2009). Urban areas were defined in the BYP as small areas with a population of 10,000 people or more using an indicator developed to categorise small areas as broadly urban or rural (ISER, 2008). The 10,000 threshold was a common definition of urbanicity for England and Wales and for Scotland. England and Wales had a different set of more graded definitions of urbanicity than Scotland and so these could not be combined further. A similar indicator developed by Gonthier (2009) was used in the NLSCY. This dichotomised census subdivisions (relatively large areas) into those with a population of 10,000 and those with fewer than 10,000.

#### Geographical region

The possibility that reporting of low self-esteem might vary broadly within countries was adjusted for by including region (Britain) or province of residence (Canada). In Britain, Scotland and Wales were included as ‘regions’. In Canada four provinces which had small sample numbers, Newfoundland, Prince Edward Island, Nova Scotia and New Brunswick, were combined into a ‘Maritimes’ region.

## Statistical analyses

The analyses were conducted in Stata (version 10, described by StataCorp, 2007). We included individuals in the survey for whom we had complete data at two time points (sample derivation described in Fig. 1), a decision made to allow a consistent sample across analyses. Comparison of those who were excluded with those who were included in samples did not highlight significant systematic biases on the analysis variables (analyses not reported here).

We describe outcome and other time varying variables by age and sex using survey weighted means and proportions with 95% confidence intervals (Table 1). Those variables which did not vary with age or sex over this period (such as family structure or neighbourhood deprivation) were described using the same method for girls aged 11 years. To allow for the complex sample designs of the surveys and for the effects of attrition in the NLSCY, we employed the ‘svyset’ command in Stata to weight the sample to make them more representative of the populations from which they were drawn originally.

Three level multilevel models were used to adjust for clustering of repeat measures of self-esteem within individuals, and individuals within DAs (NLSCY) and wards/postcode sectors (BYP). Thus repeated measures constituted the first level of the model, individuals the second level and small areas, the third level. Models were fitted using the ‘xtmelogit’ command.

To examine relationships between neighbourhood deprivation and self-esteem we first examined if neighbourhood deprivation was associated with low self-esteem before and after adjustment for individual and family characteristics across the whole sample.

We examined whether the association between neighbourhood deprivation and low self-esteem varied by age, sex, maternal education, household income, family structure and urban/rural status by including interaction terms and then by examination of stratified models. We also examined interactions by sex and age with other covariates following extensive previous work on self-esteem which suggests that associations between self-esteem and family level covariates may vary by both these variables. Finally, as relationships between neighbourhood deprivation and the odds of reporting low self-esteem differed by sex (as suggested by interactions in models), we present our final models separately for boys and girls.

Model coefficients are expressed as odds ratios (OR), and statistically significant associations are indicated where the 95% confidence interval does not include 1.00. Differences between models in terms of fit were assessed using Akaike's Information Criteria (AIC) (Akaike, 1974) and random variation at the individual and neighbourhood level was presented using the median odds ratio to allow comparison of the relative importance of these terms with odds ratios of fixed effects of neighbourhood deprivation (Merlo et al., 2006).

## Results

Table 1 shows that trends in self-esteem, parenting and peer perceptions of Canadian and British boys and girls are similar. Low self-esteem becomes more prevalent for girls than boys with increasing age, a widening gender gap that is observed in both countries. In addition to self-esteem, perceptions of relationships with peers and family appear to interact in complex ways with age and sex in both the NLSCY and the BYP.

Table 2 shows the samples by their composition in terms of family and neighbourhood characteristics which were invariant with age and sex. The results show that the NLSCY generally represents national levels of neighbourhood deprivation (there is approximately 20% of the sample in each quintile), while the BYP under-represents those living in the least deprived areas. There are comparable proportions by family structure, household income, and urbanicity in both samples. There are differences in proportions between the two countries: fewer mothers report levels of symptoms classified as distress in the NLSCY than in the BYP; lower proportions of mothers in the NLSCY were less well educated than in the BYP, but it is important to remember that these are measured differently.

We conducted sensitivity tests for hierarchical structures in the variation of self-esteem, shown in Electronic Appendix 3, available with the online version of the paper. The median odds ratio (MOR) for the between-individual variation in low self-esteem was relatively high for both samples (NLSCY MOR = 4.81, BYP MOR = 4.60). In contrast, the MORs for the between-neighbourhood NLSCY and BYP are considerably smaller at 1.33 and 1.82 respectively. This is due to the small number of individuals residing in each neighbourhood unit. The AIC increased in both the NLSCY and BYP three level model compared to the two level model, suggesting that using a more complicated (three level) model did not increase the fit to the data. Further models presented were run with adjustment for random variation at the individual level only.

Table 3 shows unadjusted and adjusted associations between individual and family level variables and the odds of reporting low self-esteem. Demographically, the odds of reporting low self-esteem are higher for girls and older children. The interaction terms – not reported here – also confirm the widening of the sex difference in self-esteem by increasing age in both samples. There were no differences by visible minority status in either sample. Relationships between household mediators were similar across both samples both before and after adjustment. Adolescent-perceived social interaction variables (perceptions of relationships with parents and friends) were protective of low self-esteem before and after adjustment. While reconstituted family structure was independently associated with the odds of reporting low self-esteem for boys in both samples and for girls in Britain, adolescents living with single parent families were not more likely to have low self-esteem than those living with both biological parents in either the NLSCY or the BYP. Maternal depression constituted a risk for adolescent self-esteem for girls in both samples (marginally non-significant in the NLSCY) but not for boys.

The relationships between family level socio-economic status as measured by household income and maternal education are quite different before and after adjustment (see Table 4). While both variables are associated with the odds of reporting low self-esteem before adjustment (in both samples), the relationship with income is largely attenuated following inclusion of the block of more socially oriented variables such as parenting and friendship perceptions (intermediary model not presented here) and differences only remain significant for the protective association between high income and low self-esteem for boys in Britain. Associations with maternal education were similar for both samples, and suggested that unadjusted protective associations with increasing levels of education were attenuated by social factors in the family such as parent–child relationships.

Table 4 presents the results findings for the relationship between neighbourhood deprivation and low self-esteem for both samples. Unadjusted odds ratios suggested that there were no relationships between adolescent self-esteem and neighbourhood deprivation in the BYP and the NLSCY. These findings were unchanged by adjustment for individual and family variables except that a protective association was found for quintiles 3 and 4, the odds of reporting low self-esteem in those areas was *lower* in these more deprived quintiles than in the least deprived quintiles. A significant interaction was found between neighbourhood deprivation and sex in the NLSCY, suggesting that the relationship between deprivation and low self-esteem varied for boys and girls. Following this finding, models were stratified by sex for both samples.

The sex stratified models showed a slight divergence in the neighbourhood deprivation findings between the NLSCY and the BYP. The adjusted odds for girls remained non-significant in the NLSCY. In contrast, the odds of reporting low self-esteem were protective for the more deprived quintiles (2–5) for boys. This was interpreted as evidence of a threshold effect, whereby adolescents were *more* likely to report low self-esteem in the least deprived 20% of neighbourhoods than in the other 80% of neighbourhoods. Interactions suggested that these sex-specific findings in the NLSCY were not moderated by urbanicity, family structure or household income. Results in the BYP were non-significant in the sex-stratified models (see Table 4).

## Discussion

The socio-economic equalisation in youth hypothesis was specified as a life-course hypothesis and should be tested with longitudinal data from early childhood, through adolescence to adulthood (West, 1997). Our data pertain to the adolescent stage only; we therefore put forward this evidence as supporting the equalisation hypothesis at this stage of the life course, and not as a direct test of the hypothesis as fully specified. We suggest that as the adolescents analysed here are now adults, further research with these datasets might be used to test this hypothesis more directly.

Our findings have significance for international debates about the importance of neighbourhood deprivation for adolescent self-esteem and wellbeing. With respect to our first aim, concerning equality of health during adolescence, we find support for socio-economic equalisation of self-esteem across levels of neighbourhood deprivation in Britain and Canada. In Britain, we find that the risk of reporting low self-esteem is no different in deprived neighbourhoods compared to the least deprived neighbourhoods. In Canada, we find that the risk of reporting low self-esteem may be higher in the least deprived neighbourhoods than in the most deprived. We could not identify a clear reason for this apparently higher prevalence of low self-esteem in the least deprived neighbourhoods in Canada. There are many possible substantive and methodological reasons why this may have arisen and these might be pursued if this finding is replicated in other studies. Moreover, we did find that self-esteem, whilst unstable in adolescents over time (as suggested by the large median odds ratios reported in Tables 3 and 4), did not vary substantially between neighbourhoods at the geographical scales of analysis that we investigated.

These findings are supported by nationally representative work in Britain which has investigated unadjusted associations between deprivation and self-reported emotional problems (Collishaw et al., 2009). They are also supported by regional studies investigating malaise symptoms (West & Sweeting, 2004) and psychological distress (Fagg et al., 2006). Our findings extend this small evidence base for socio-economic equalisation at the area level in Britain, to low self-esteem, and to Canadian adolescents.

We note that our findings contrast with a nationally representative study which examines the association between neighbourhood deprivation and adolescent self-esteem in the United States (Turley, 2002). They also contrast with another U.S. study which investigates the association between depressive symptoms and deprivation (Wickrama & Bryant, 2003). These studies tended to support the case for neighbourhood deprivation as an independent factor relating to psychological outcomes for adolescents.

While testing the socio-economic equalisation hypothesis in relation to family level was not a central objective of our study, it is important to note that our findings were not consistent with previous work from Britain cited in support of the equalisation hypothesis (West, 1997; West & Sweeting, 2004). We found unadjusted associations between household income and maternal education (markers of socio-economic status) and low self-esteem, suggesting that there were socio-economic inequalities at the family level. These findings were also replicated in the NLSCY. In both studies, adjustment for adolescents' perceptions of friends, family and parent–adolescent relationships generally attenuated these relationships to non-significance. This may indicate that socioeconomic status has an association with self-esteem which is mediated by personal relationships for this sample.

With respect to our second aim, the results suggest that self-esteem is equalised by neighbourhood deprivation for both boys and girls though in Canada the relationships vary by sex. There is no evidence that the relationship between neighbourhood deprivation and self-esteem varies by family socio-economic status, family structure, maternal education, or urban/rural status. This suggests that the support for the equalisation hypothesis is consistent within these socio-demographic groups in both Britain and Canada.

Finally, we find that patterns of association for Canada and Britain are similar to each other, but do not seem to correspond with published nationally representative findings from other research in the United States. This strengthens the evidence base suggesting that there is a substantive national difference in the relationship between neighbourhood deprivation and low self-esteem in between the United States and Britain. The relationship between neighbourhood deprivation and low self-esteem found in our analysis for Canada is also different from that reported in other research from the United States.

Adolescents and their families in the Canadian and British context benefit from state-subsidised health and social care systems whereas in the United States these systems are less well established. Added to this, social determinants of health such as income inequality, absolute poverty and crime in the neighbourhood are more pronounced in the United States than in either Canada or Britain (Marmot, 1998; Oreopoulos, 2008). Thus, living in a relatively poor neighbourhood may well be qualitatively different for an adolescent in the United States when compared to an adolescent in either Britain or Canada.

Our findings relating to adolescent self-esteem may also be considered in relation to studies examining the relationship between neighbourhood deprivation and depression; national differences between samples in Britain and the United States have been observed in a systematic review of the literature (Mair et al., 2008). More broadly these national differences have been observed in work comparing rates of mortality in Canada and the United States by income inequality (Ross et al., 2000). We suggest that there is a case for extending these types of cross-country comparisons for adolescence, ideally using study designs which can test these national level differences in associations quantitatively.

There are limitations to our analysis. Both our measures of self-esteem were self-reported, and were dichotomised using statistical cut-offs. This means that we cannot be sure how far the outcomes used here have construct validity as measures of low self-esteem. However, to our knowledge there are no other sources of nationally representative data in either Canada or Britain which measure adolescent self-esteem. Furthermore, our findings from both countries are broadly consistent with previous work in terms of associations with age and sex and family covariates (Baldwin & Hoffmann, 2002). Finally, for British data, which were closer to a normal distribution, we tested growth curve models (not reported here). There was no association between deprivation and self-esteem, illustrating that the loss of information through dichotomisation did not underlie the lack of relationship reported here.

Our measures of neighbourhood deprivation are broad, raising the possibility that no relationship was found due to measurement error. However, it is important to note that previous work has established the presence of area level socio-economic inequalities in health for adults using the same measures (Curtis, Setia, & Quesnell-Vallee, 2009; Townsend, Davidson, & Whitehead, 1988). In addition, as mentioned above, differences in the geographical scale of deprivation indices and that they were measured 10 years apart may have explained differences and similarities between findings from Britain and Canada. We conducted sensitivity analyses (results not reported) with indices of deprivation measured at different geographical scales (census tracts in Canadian urban areas, lower super output areas in England and Wales (not available for Scotland)) and different time-points (in analyses at ward level and lower super output area level in England where these data were measured in 2001). Findings from these analyses supported our conclusions; none of the above mentioned deprivation indices were associated with low self-esteem in a way indicative of a socio-economic gradient at the small area level.

Our analysis has important strengths. We used large national datasets with the potential to generalise estimates to the adolescent population of British and Canada. This also meant that we had sufficient power and variability to test whether deprivation was associated with our outcome across key moderators which varied across the population (such as urban/rural status).

## Conclusion

Our paper has broad implications for the ways that neighbourhood research is used to inform policy. Policy makers should be aware that the effects of neighbourhood-based policies and programmes may not be equally important for all health outcomes. Adolescent self-esteem appears rather equal across neighbourhoods with varying levels of deprivation in the two countries examined. This may mean that in Britain and Canada, resources to address problems of low adolescent self-esteem and wellbeing might be most effectively directed towards programmes at the family or school level and which support positive parent–child relationships, through a strategy of ‘progressive universalism’, supporting disadvantaged adolescents in all geographical settings (Marmot et al., 2010), rather than targeting whole neighbourhoods that are relatively deprived. In contrast, other research reviewed above suggests that neighbourhood deprivation may be an important factor for self-esteem at this stage in the life course in the United States. Therefore it should not be assumed that patterns of inequality found in samples from one country will necessarily be applicable to other national contexts and this is suggestive of an interesting research agenda concerning the significance of national contexts for adolescent psychological wellbeing.

Finally, while we focused our analyses on low self-esteem the analysis of other mental health outcomes are important, but were beyond the scope of this paper. Further work could extend analyses of these data to compare effects on depressive symptoms in the UK and Canada which, like self-esteem, are measured differently but are similar in terms of the latent constructs that they aim to capture.

## Figures and Tables

**Table 1:** Population weighted means, proportions and 95% confidence intervals of outcomes and covariates by age and sex for time-varying variables in the NLSCY (*N* = 3421 overall, see top part of table) and BYP (*N* = 1927 overall, see lower part of table). BYP data are presented for ages 11,13 and 15 years (sample also ages 12 and 14 years). Age and sex trends are similar for full and reduced presentation, and the reduced data are presented here to facilitate interpretation and comparison with the NLSCY.

Age	NLSCY girls (*N* = 1773, 5192 observations)			NLSCY boys (*N* = 1648, 4850 observations)		
	10/11 yrs (*n* = 1755)	12/13 yrs (*n* = 1773)	14/15 yrs (*n* = 1664)	10/11 yrs (*n* = 1636)	12/13 yrs (*n* = 1648)	14/15 yrs (*n* = 1566)
	Prop./mean (95% CI)	Prop./mean (95% CI)	Prop./mean (95% CI)	Prop./mean (95% CI)	Prop./mean (95% CI)	Prop./mean (95% CI)
Low self-esteem	0.10 [0.08,0.12]	0.19 [0.16,0.22]	0.25 [0.22,0.28]	0.11 [0.09,0.14]	0.15 [0.13,0.18]	0.15 [0.12,0.18]
Parental nurture (mean)	15.2 [14.9,15.5]	16.0 [15.7,16.2]	15.1 [14.8,15.4]	14.5 [14.2,14.8]	15.4 [15.2,15.7]	14.6 [14.2,14.9]
Parental rejection (mean)	5.7 [5.4,6.1]	7.65 [7.4,8.0]	8.7 [8.4,9.1]	6.7 [6.3,7.0]	8.5 [8.2,8.8]	9.5 [9.1,9.9]
Friendship quality (mean)	13.0 [12.8,13.2]	13.55 [13.4,13.7]	13.6 [13.4,13.8]	12.3 [12.0,12.5]	12.7 [12.5,12.9]	13.3 [13.1,13.5]
Family functioning (mean)	7.6 [7.5,7.8]	7.7 [7.5,7.8]	7.5 [7.4,7.6]	7.8 [7.7,7.9]	7.6 [7.5,7.7]	7.5 [7.4,7.6]
	BYP Girls (*N* = 979, 4101 observations across *all* ages)			BYP Boys (*N* = 948, 3961 observation across *all* ages s)		
Age	11	13	15	11	13	15
Variables	Prop [95% CI]	Prop [95% CI]	Prop [95% CI]	Prop [95% CI]	Prop [95% CI]	
Low self-esteem	0.08 [0.06,0.10]	0.12 [0.10,0.14]	0.15 [0.12,0.19]	0.08 [0.06,0.10]	0.07 [0.05,0.09]	0.05 [0.03,0.07]
*Talks to parent about ‘close things’*						
Hardly ever	0.51 [0.46,0.55]	0.43 [0.40,0.47]	0.37 [0.33,0.42]	0.52 [0.48,0.57]	0.49 [0.45,0.52]	0.47 [0.43,0.51]
Regularly	0.38 [0.34,0.42]	0.44 [0.40,0.48]	0.48 [0.44,0.53]	0.38 [0.34,0.42]	0.43 [0.39,0.47]	0.45 [0.41,0.49]
Most days	0.12 [0.09,0.14]	0.12 [0.10,0.15]	0.14 [0.11,0.17]	0.10 [0.07,0.12]	0.08 [0.06,0.11]	0.08 [0.06,0.10]
*Argues with parent*						
Hardly ever	0.18 [0.15,0.21]	0.22 [0.20,0.25]	0.23 [0.20,0.27]	0.26 [0.22,0.29]	0.39 [0.35,0.42]	0.36 [0.32,0.40]
Regularly	0.38 [0.34,0.42]	0.41 [0.37,0.44]	0.42 [0.38,0.47]	0.40 [0.36,0.44]	0.42 [0.38,0.46]	0.49 [0.45,0.53]
Most days	0.44 [0.40,0.48]	0.37 [0.33,0.40]	0.34 [0.30,0.39]	0.35 [0.31,0.39]	0.2 [0.17,0.23]	0.15 [0.12,0.18]
*Friendship Happiness (ref. happy)*						
Unhappy with friends	0.04 [0.03,0.06]	0.06 [0.04,0.07]	0.07 [0.05,0.09]	0.06 [0.04,0.08]	0.05 [0.03,0.07]	0.03 [0.01,0.04]
*Family Happiness (ref. happy)*						
Unhappy	0.05 [0.03,0.06]	0.07 [0.05,0.09]	0.11 [0.08,0.14]	0.05 [0.03,0.06]	0.07 [0.05,0.09]	0.11 [0.08,0.14]

**Table 2:** Population weighted means and proportions and 95% confidence intervals of age and sex invariant variables in the NLSCY (*N* = 3421) and BYP (1927).

NLSCY		BYP	
Variables	Girls aged 10/11	Variables	Girls aged 11
*Race/ethnicity*		*Race/ethnicity*	
White European heritage	0.85 [0.71,0.79]	White European heritage	0.95 [0.93,0.97]
First nations	0.10 [0.07,0.12]	Visible minority	0.05 [0.03,0.07]
Visible minority	0.05 [0.03,0.07]		
*Family structure*		*Family structure*	
Intact	0.75 [0.72,0.79]	Intact	0.70 [0.66,0.73]
Reconstituted	0.09 [0.07,0.11]	Reconstituted	0.12 [0.10,0.15]
Single parent	0.15 [0.12,0.19]	Single parent	0.18 [0.15,0.21]
*Household income*		*Household income*	
Average	0.53 [0.49,0.57]	Average	0.50 [0.46,0.54]
High	0.30 [0.27,0.34]	High	0.30 [0.26,0.33]
Low	0.17 [0.14,0.20]	Low	0.20 [0.17,0.24]
*Maternal depression*		*Maternal depression*	
Not depressed	0.82 [0.79,0.86]	Not depressed	0.69 [0.65,0.73]
Depressed	0.18 [0.14,0.21]	Depressed	0.31 [0.27,0.35]
*Maternal education*		*Maternal education*	
<High school	0.21 [0.18,0.25]	< GCSE	0.36 [0.32,0.40]
High school	0.63 [0.59,0.67]	GCSE	0.32 [0.28,0.36]
Diploma or Degree	0.16 [0.13,0.18]	A-level	0.09 [0.07,0.11]
		Diploma or Degree	0.24 [0.21,0.28]
*Neighbourhood deprivation*		*Neighbourhood deprivation*	
Least deprived (Q1)	0.19 [0.15,0.22]	Least deprived (Q1)	0.11 [0.08,0.13]
Q2	0.20 [0.17,0.23]	Q2	0.17 [0.14,0.20]
Average	0.23 [0.20,0.27]	Average	0.25 [0.21,0.28]
Q4	0.20 [0.17,0.23]	Q4	0.26 [0.22,0.30]
Most deprived (Q5)	0.18 [0.15,0.21]	Most deprived (Q5)	0.21 [0.18,0.25]
*Urbanicity*		*Urbanicity*	
Rural	0.23 [0.20,0.25]	Rural	0.24 [0.21,0.28]
Urban	0.77 [0.75,0.80]	Urban	0.76 [0.72,0.79]
*Province*		*Region*	
Maritimes	0.07 [0.06,0.08]	Midlands/East of England	0.21 [0.18,0.25]
Quebec	0.27 [0.23,0.30]	London	0.09 [0.07,0.12]
Ontario	0.38 [0.34,0.42]	North West of England	0.10 [0.07,0.12]
Manitoba	0.03 [0.02,0.04]	North of England	0.13 [0.10,0.16]
Saskatchewan	0.04 [0.03,0.04]	Northern Ireland	0.00 [0.00,0.00]
Alberta	0.10 [0.08,0.12]	Scotland	0.07 [0.05,0.09]
British Columbia	0.11 [0.09,0.13]	South of England	0.33 [0.29,0.37]
		Wales	0.06 [0.04,0.07]

**Table 3:** Individual and family level associations with low self-esteem in early adolescence.

NLSCY (*N* = 3421)			BYP (*N* = 1927)		
NLSCY variables	Unadjusted	Adjusted	BYP variables	Unadjusted	Adjusted
	O (95% CI)	O (95% CI)		O (95% CI)	O (95% CI)
*Age (ref. 10/11 yrs)*			*Age (ref. 11)*		
12/13 yrs.	2.20 [1.87,2.59]	2.34 [1.84,2.98]	12	1.26 [0.95,1.68]	1.13 [0.84,1.52]
14/15 yrs.	2.67 [2.26,3.15]	2.33 [1.75,3.11]	13	1.16 [0.88,1.53]	0.97 [0.72,1.32]
*Sex (ref. boys)*			14	1.42 [1.06,1.89]	1.13 [0.82,1.55]
Girls	1.59 [1.34,1.88]	2.92 [2.40,3.54]	15	1.31 [0.97,1.77]	1.03 [0.73,1.45]
*Ethnicity (ref. White European)*			*Sex (ref. Boys)*		
First Nations	1.02 [0.74,1.41]	0.93 [0.65,1.32]	Girls	2.20 [1.71,2.84]	2.11 [1.65,2.69]
Other Visible Minority	1.34 [0.87,2.07]	0.89 [0.56,1.41]	*Ethnicity (ref. Non-visible minority)*		
Parental Nurture	0.79 [0.78,0.81]	0.81 [0.79,0.83]		0.97 [0.50,1.88]	0.93 [0.49,1.74]
Parental Rejection	1.19 [1.16,1.21]	1.12 [1.09,1.14]	*Talks with parents (ref. ‘Regular’)*		
Friendship Quality	0.74 [0.72,0.76]	0.72 [0.69,0.74]	Hardly ever	1.31 [1.04,1.66]	1.35 [1.06,1.72]
Family Functioning	0.88 [0.83,0.93]	1.00 [0.94,1.07]	Most days	0.54 [0.40,0.75]	0.67 [0.48,0.93]
*Family structure (ref. Intact)*			*Argues with parents (ref. ‘Regular’)*		
Reconstituted	1.86 [1.46,2.38]	1.50 [1.15,1.97]	Hardly ever	0.45 [0.36,0.58]	0.56 [0.43,0.71]
Single Parent	1.69 [1.37,2.09]	1.07 [0.83,1.39]	Most days	2.02 [1.44,2.81]	1.58 [1.12,2.23]
*HH income (ref. Average)*			*Friend happiness (ref. Happy)*		
High	0.73 [0.61,0.88]	0.90 [0.72,1.12]	Unhappy	6.57 [4.74,9.09]	5.27 [3.79,7.33]
Low	1.32 [1.09,1.59]	1.11 [0.88,1.40]	*Family happiness (ref. Happy)*		
*Maternal education (ref. < High school)*			Unhappy with family	6.30 [4.68,8.48]	4.25 [3.14,5.75]
High school	0.77 [0.63,0.94]	0.87 [0.69,1.09]	*Family structure (ref. Intact)*		
Diploma or Degree	0.54 [0.40,0.72]	0.66 [0.48,0.91]	Reconstituted	2.04 [1.51,2.76]	1.74 [1.30,2.33]
*Maternal depression (ref. Not depressed)*			Single Parent	1.61 [1.19,2.18]	1.10 [0.80,1.51]
Depressed	1.44 [1.19,1.74]	1.17 [0.94,1.47]	*HH income (ref. Average)*		
			High	0.63 [0.49,0.82]	0.65 [0.50,0.86]
			Low	1.33 [1.04,1.70]	1.22 [0.95,1.58]
			Maternal education (ref. <GCSE)		
			GCSE	0.77 [0.57,1.04]	0.87 [0.65,1.16]
			A-level	0.70 [0.44,1.11]	0.85 [0.55,1.33]
			Diploma or degree	0.62 [0.44,0.87]	0.70 [0.50,0.97]
			*Maternal depression (ref. Not depressed)*		
			Depressed	1.51 [1.24,1.85]	1.42 [1.16,1.74]
Individual level MOR	n/a*	5.01		n/a	4.12

* Unadjusted odds and 95% confidence intervals were calculated with a separate multilevel model for each variable (hence MORs not reported). Adjusted odds were calculated following adjustment for all individual and family variables and measurement year (not shown here).

**Table 4:** Unadjusted and adjusted models of low self-esteem and neighbourhood deprivation over full samples in the NLSCY and BYP.

Covariates	Unadjusted	Adjusted^a^	Girls – adjusted^a^	Boys – adjusted^a^
	OR [95% CI]	OR [95% CI]	OR [95% CI]	OR [95% CI]
*BYP models*				
*Neighbourhood deprivation*				
Quintile 1 (least deprived)	1	1	1	1
2	1.20 [0.71,2.02]	1.10 [0.66,1.82]	0.95 [0.50,1.79]	1.47 [0.62,3.50]
3	1.55 [0.95,2.52]	1.19 [0.73,1.94]	1.22 [0.67,2.23]	1.26 [0.53,2.98]
4	1.45 [0.90,2.34]	1.18 [0.71,1.96]	1.05 [0.56,1.99]	1.47 [0.62,3.49]
Quintile 5 (most deprived)	1.49 [0.92,2.42]	1.25 [0.74,2.13]	1.08 [0.56,2.10]	1.65 [0.67,4.09]
*Model statistics*				
N	1927	1927	979	948
AIC		4356	2607	1775
Median odds ratio		4.04	3.86	4.03
*NLSCY models*				
N	3421	3421	1773	1648
*Neighbourhood deprivation*				
Quintile 1 (least deprived)	1	1	1	1
2	0.95 [0.71,1.28]	0.81 [0.59,1.13]	1.03 [0.67,1.59]	0.60 [0.36,0.98]
3	0.94 [0.71,1.26]	0.68 [0.49,0.93]	0.79 [0.51,1.21]	0.53 [0.32,0.88]
4	0.94 [0.70,1.25]	0.63 [0.45,0.87]	0.82 [0.53,1.26]	0.45 [0.27,0.76]
Quintile 5 (most deprived)	1.00 [0.76,1.33]	0.72 [0.51,1.01]	0.94 [0.60,1.48]	0.50 [0.30,0.86]
*Model statistics*				
N	3421	3421	1773	1648
AIC		5888	2539	3344
Median odds ratio		3.62	1.80	1.83

a Models adjusted age and sex interaction, visible minority status, adolescents' parenting, family and peer group perceptions, household income, family structure, province/region, and urbanicity.
