# Supplementary material for: Neighbourhood deprivation and adolescent self-esteem: Exploration of the ‘socio-economic equalisation in youth’ hypothesis in Britain and Canada
Source: Soc Sci Med. 2013 Aug;91:168–77. doi: 10.1016/j.socscimed.2013.02.021 (PMC3726937; doi:10.1016/j.socscimed.2013.02.021)
Supplement: Supplementary file 1 [file mmc1.doc]

ELECTRONIC APPENDIX 1: Comparison of the NLSCY and BYP scales with Rosenberg self-esteem scale

Both scales draw conceptually on the Rosenberg scale (Rosenberg, 1965) but are operationalised differently. All four of the NLSCY items map directly on to the Rosenberg scale, while one item of the BYP does not. In addition, and perhaps more importantly, the NLSCY items are all positively worded. This means that responses to the scale may be positively biased by the tendency of some respondents to answer all questions in a similar way. This bias is potentially averaged out in the mixed valence BYP and Rosenberg scales. Overall, although both scales are different, they do represent the Rosenberg scale conceptually, measuring global self-esteem. Perhaps most importantly, both short form scales contain items measuring self-worth (e.g. I like the way I am) and competence (e.g. I have a lot to be proud of) indicating that they are conceptually similar even in short form.

| **Rosenberg 10 item scale** | **NLSCY** | **BYP** |
| --- | --- | --- |
| On the whole, I am satisfied with myself (+) | In general I like the way I am. (+) |  |
| I feel I do not have much to be proud of (-) | Overall I have a lot to be proud of (+) |  |
| I take a positive attitude toward myself (+) | A lot of things about me are good (+) |  |
| I am able to do things as well as most other people (+) | When I do something I do it well (+) |  |
| At times, I think I am no good at all (-) |  | At times I feel I am no good at all (-) |
| I feel that I’m a person of worth, at least on an equal plane with others (+) |  |  |
| I wish I could have more respect for myself (+) |  | I am a likeable person (+) |
| All in all, I am inclined to feel that I am a failure (-) |  | I am inclined to feel I am a failure (-) |
| I feel that I have a number of good qualities |  | I feel I have a number of good qualities (+) |
| I certainly feel useless at times (-) |  | I certainly feel useless at times (-) |
| (+) indicates that the question has a positive valence, (-) indicates that the valence is negative. | | |

ELECTRONIC APPENDIX 2: Comparison of the NLSCY and BYP parenting and peer measurements

#### Adolescent perceptions of parents

The BYP measured four items measuring how frequently adolescents perceived that they argued with their mother or father, or talked about close things, again with each parent separately. All items were measured similarly and ranged from 1 to 5. The values represented for each of the four items: 1 - hardly ever, 2 - less than once a week, 3 - more than once a week, 4 – most days, and 5 – no mother / father (depending on whether the item referred to the mother or the father).

Using these four items, two variables were derived, measuring frequency of talking about close things and frequency of arguments. These were both derived in the same way. Where responses were available between 1 and 4 for the items referring to the mother and the father, the most positive response was taken. Thus, for the talking variable this would be the highest value (i.e. assuming that talking about close matters frequently is most desirable). For the arguments variable, this would be the lowest value (i.e. assuming that arguing infrequently with parents is more desirable). Where no response was available (as in single parent families or where the response was 5- no mother / father), the response about the other parent was used. The middle categories of more than once a week and less than once a week were then collapsed to a single category of ‘regular’. This was done to allow the most clearly conceptualised categories (of hardly ever or most days) to be compared with a single category which lay between them.

The NLSCY utilised two multi-item parenting scales corresponding to nurturing and rejection. These were developed from a factor analysis of an inventory of parenting questions developed by Schaefer et al. (1965). These scales were derived from adolescents’ responses to statements starting with “My parents (or step parents or foster parents or guardians).

The nurturance scale (theoretical range = 6-30, Cronbach’s alpha = 0.77) was made up of 6 items. The items are listed below. The rejection scale (range = 7-35, Cronbach’s alpha = 0.59). All items were scored using a 5 point likert scale by the adolescent.

Figure 3‑3: Items in parental nurturance and rejection scales in the NLSCY

| **Nurturance Scale** | **Rejection scale** |
| --- | --- |
| …smile at me | ….soon forget a rule they have made |
| …praise me | …nag me about little things |
| …make sure I know I am appreciated | … only keep rules when it suits them |
| …seem proud of the things I do | … threaten punishment more often than they use it |
| ….listen to my ideas and opinions | …enforce a rule or do not enforce rule depending on their mood |
| …and I solve a problem together whenever we disagree about something | …hit me or threaten to do so |
|  | …get angry and yell at me |
| Where the possible responses to the items in both scales were: 1, Never; 2, Rarely; 3, Sometimes; 4, Often; 5, Always; 6, Not Applicable; 7 Don’t know; 8, Refusal; 9, Not Stated. Items 6-9 were recoded in analyses as *system missing*. | |

#### Relationships with friends and peers

In the BYP peer relationships were assessed using a single item which captured whether the respondent was ‘happy with their friends’ with possible responses ranging from 1-completely happy, 2, 3, 4-don’t know, 5, 6, 7-unhappy. These categories were conflated to happy and ‘don’t know or unhappy’ owing to the very small proportions who reported unhappiness with family (only 3% reported any unhappiness (5-7) with friends at all).

In the NLSCY, peer relationships are operationalised using the peer relations subscale (Marsh and O'Neil, 1984). These items are self-reported by 10-15 year olds in Cycles 1-5. The items making up the scale are made up of the following statements by the adolescent: ‘I have many friends’, ‘I get along with others my age’, ‘Others my age want me to be their friend’, ‘Most others my age like me’. These statements are then assessed by the adolescent with respect to the following possible responses - 1, False; 2, Mostly False; 3, Sometime true/Sometimes False; 4, Mostly true; 5, True. The scale ranges from 0-16, where 0 indicates that the respondent does not have positive relationships, or have many friends. While the first question measures the number of friends as opposed to the degree to which the respondent gets on with others, the scale appears to be internally consistent with Cronbach’s alpha scores of 0.78 (StatCan, 2006).

Family functioning has been found consistently to be associated with mental health in young children and adolescents and may vary by neighbourhood type and with young people’s mental health (Leventhal and Brooks-Gunn, 2000).

Family functioning was measured in the BYP by asking the adolescent the question to rate how they feel about their family on a scale of one to seven where feelings were indicated by a series of “faces expressing various types of feelings” (Taylor, 2009). This was the closest proxy that could be found which was available at all waves of the BYP.

Family functioning was measured in the NLSCY using the 12-item general subscale of the McMaster Family Assessment Device (Epstein, Baldwin and Bishop, 1983). The statements describe family behaviour and relationships and measure problem solving, communication between family members, family roles, and affective relationships within the family. The ‘Person Most Knowledgeable about the child (PMK: see glossary) responded on a on a scale ranging from 1, strongly agree to 4, strongly disagree. Negatively coded responses were reverse coded, 1 was subtracted from each item, and the items then summed. Scores range from 0 to 36, with higher scores representing greater family functioning. The alpha coefficient for this score was calculated as 0.86 over the sample.

ELECTRONIC APPENDIX 3: Two and three level variance components models for NLSCY and BYP

|  | **NLSCY (n=3421)** | | **BYP (n=1927)** | |
| --- | --- | --- | --- | --- |
| **Parameters** | **M1a: 2 level** | **M1b: 3 level** | **M2a: 2 level** | **M2b: 3 level** |
| **Variance (standard error)** |  |  |  |  |
| Neighbourhood | … | 0.087 (0.151) | … | 0.39 (0.21) |
| Adolescent | 2.821 (0.272) | 2.73 (0.309) | 2.30 (0.95) | 2.56 (0.35) |
| **Median Odds Ratios** |  |  |  |  |
| Neighbourhood | … | 1.33 | … | 1.82 |
| Adolescent | 4.96 | 4.84 | 5.21 | 4.60 |
| **Intra-class correlations** |  |  |  |  |
| Neighbourhood | … | 7% | … | 6% |
| Adolescent | 43% | 37% | 48% | 41% |
| **Fit** |  |  |  |  |
| Log likelihood | -3675 | -3675 | -2404 | -2402 |
| AIC** | 7434 | 7436 | 4812 | 4810 |
| Lrtest* against null model | Chi = 361, p<0.001 | Chi = 361, p < 0.001 | Chi = 354,  p <0.001 | Chi = 358,  p < 0.001 |
| Lrtest* against Ind. model | … | Chi = 0.65, p =0.421 | … | Chi = 4,  p=0.021 |
| Neighbourhood  N (min, mean, max) | … | 1958 (1, 4, 200) | … | 851 (2,9,62) |
| Adolescents N (min, mean, max) | 2432 (2,3,3) | 2432 (2,3,3) | 1927 (2,4,5) | 1927(2,4,5) |
| *Where ‘lrtest’ refers to the likelihood ratio test and tests statistical significance of differences between models. ** AIC refers to Akaike’s Information Criteria and tests improvements in fit over and above increases in complexity. | | | | |
